# Diversity and Plant Growth-Promoting Properties of *Rhodiola rosea* Root Endophytic Bacteria

**DOI:** 10.3390/microorganisms13010013

**Published:** 2024-12-25

**Authors:** Inga Tamošiūnė, Muhammad Fahad Hakim, Odeta Buzaitė, Vidmantas Stanys, Jurgita Vinskienė, Elena Andriūnaitė, Danas Baniulis

**Affiliations:** 1 Institute of Horticulture, Lithuanian Research Centre for Agriculture and Forestry, Kaunas Str. 30, 54333 Babtai, Kaunas reg., Lithuania; inga.tamosiune@lammc.lt (I.T.); fahad.hakim@lammc.lt (M.F.H.); vidmantas.stanys@lammc.lt (V.S.); jurgita.vinskiene@lammc.lt (J.V.); elena.andriunaite@lammc.lt (E.A.); 2 Department of Biochemistry, Vytautas Magnus University, Universiteto Str. 10, 53361 Akademija, Kaunas reg., Lithuania; odeta.buzaite@vdu.lt

**Keywords:** metataxonomic analysis, microbiome, plant-associated bacteria, rhizome, succulent plant

## Abstract

Plants inhabiting environments with suboptimal growth conditions often have a more pronounced capacity to attract and sustain microbial communities that improve nutrient absorption and expand abiotic stress tolerance. *Rhodiola rosea* L. is a succulent plant of the *Crassulaceae* family adapted to survive in sandy or rocky soils or dry tundra. The aim of the present study was to investigate the diversity and plant growth-stimulating potential of *R. rosea* endophytic microbiota. Metataxonomic analysis of the bacterial diversity in the rhizome of *R. rosea* revealed 108 families. Among these, three families were found exclusively in the core microbiome of 1-year-old plants, while nine families were unique to the core microbiome of mature plants grown in the field for more than 4 years. Seventy-three endophytic bacteria isolates were obtained from the rhizome of *R. rosea* plants and were assigned into 14 distinct bacterial genera of Firmicutes (26%) or Proteobacteria (74%) phyla. Screening for functional genes related to the nitrogen cycle, phosphorus mineralisation or dissolution, and traits associated with nitrogen fixation (56% of isolates), siderophore production (40%), inorganic phosphorus solubilisation (30%), and production of indole-related compounds (51%) led to the classification of the isolates into 16 distinct clusters. Co-cultivation of 45 selected isolates with germinating Arabidopsis seedlings revealed 18 and 5 isolates that resulted in more than a 20% increase in root or shoot growth, respectively. The study results established the complexity of the succulent *R. rosea* endophytic microbiome and identified isolates for potential plant growth-stimulating applications.

## 1. Introduction

From the evolutionary perspective, establishing a mutualistic partnership between plants and microorganisms has been essential for the colonisation of terrestrial habitats by land plants [1]. The recruitment of microorganisms to expand plant metabolic and adaptive capacity, in particular, has paid off in environments with inadequate nutrient supply and suboptimal growth conditions. The relationship between the plant host and microbiota spans a broad spectrum of interactions, from association with free-living rhizosphere microbiota to apoplast-inhabiting endophytes or intracellular endosymbiosis [2,3,4]. Plant-associated microbial communities play a crucial role in the stimulation of plant growth through the absorption of nutrients or priming disease and abiotic stress tolerance [5,6,7].

Some intercellular endophytes are vertically transmitted through seeds and inhabit host plant tissues for generations [8]. Others are less adapted to a particular host and represent a diversity of microorganisms mostly related to soil microbiota [5,9]. It has been demonstrated that plants could actively modulate the composition of the endophytic microbial community by recruiting soil microorganisms [10]. Rhizodeposition of carbon sources in the form of organic acids or phenolic compounds attracts beneficial microorganisms [11]. Plants inhabiting extreme and arid environments have an enhanced capacity to enrich beneficial microbiota in the rhizosphere by root exudates and other metabolic inputs [12,13,14].

It was previously documented that plants adapted to survive under suboptimal growth conditions benefit from bacterial endophyte colonisation and could serve as a source of plant growth-promoting bacteria. Endophytic bacteria isolated from desert plants were able to promote drought tolerance or mitigate salinity stress in non-desert plants [15,16,17]. Wild plants growing in alpine regions are associated with microbial communities that may enhance cold and dehydration tolerance in host plants. Endophytic bacteria of *Duganella*, *Erwinia*, *Pseudomonas*, and *Rhizobium* spp. isolated from alpine *Rosaceae* plants improved freezing stress tolerance in strawberry [18] and common bean plants [19].

*Rhodiola* spp. belong to the *Crassulaceae* family, which includes 35 genera of mostly succulent plant species that are found worldwide, but mostly in the Northern Hemisphere and Southern Africa [20]. *R. rosea* L. is adapted to survive in sandy or rocky soils of alpine regions or dry tundra, but it is also grown throughout Europe and Asia as an ornamental and traditional medical plant [21]. Recently, Emmer et al. [22] isolated plant growth-promoting bacteria from *Echeveria laui*, a plant of the *Crassulaceae* family inhabiting seasonally dry tropical climates. Also, the diversity of endophytic fungi and their role in bioactive plant metabolite production or antioxidant activities were studied in related *R. crenulata*, *R. angusta*, *R. sachalinensis*, and *R. tibetica* species [23,24,25]. However, the diversity and plant growth-promoting potential of bacterial endophytes of alpine *Crassulaceae* species remains elusive.

The aim of the present study was to investigate the endophytic bacteria diversity of *R. rosea* plants and to assess their plant growth-stimulating potential. *16S rRNA* gene amplicon sequencing-based metataxonomic analysis was used to study the microbial diversity in the rhizome of the field-grown *R. rosea* plants. Further, we isolated culturable endophytic bacteria, and their plant growth-promoting traits were assessed using genetic and metabolic tests as well as *Arabidopsis thaliana* (L.) Heynh. seedlings.

## 2. Materials and Methods

### 2.1. Plant Sample Collection and Isolation of Cultivable Endophytic Bacteria

The *Rhodiola rosea* L. plants were grown under field conditions in a mixture of peat moss and compost, in Kaunas region, Lithuania (N 55.085480 E 23.802629). Rhizome samples were collected in the spring of 2024 from one-year-old plants initiated from seeds (1-year experimental group) and plants cultivated for at least four years (>4-year experimental group). Rhizomes extracted from the substrate were rinsed with tap water to remove excess soil. Rhizomes were then thoroughly washed three times with sterile distilled water, surface sterilised with 75% ethanol for 1 min, 5% NaOCl for 5 min, and sterile distilled water twice according to Cui et al. [24]. After the outer rhizome layer was removed, each sample was cut into 2 mm to 3 mm sections. For DNA extraction, samples were stored at −70 °C until use. For cultivable bacteria isolation, following the sterilisation procedure, the rhizome sections were placed on lysogeny broth (LB) [26] agar or actinomycete agar (Merck, Darmstadt, Germany) and incubated for 24 to 48 h, depending on bacterial colony growth, at 25 °C without light. For control of the sterilisation efficiency, intact rhizome sections were incubated on the nutrient medium after the sterilisation. The absence of bacterial growth on control plates with sterilised intact sections of rhizome indicated that the bacterial isolates originated from the inner tissues of the rhizome.

### 2.2. Plant Sample DNA Extraction, PCR Amplification, and Molecular Identification

Genomic DNA was extracted from pure bacterial culture using the GeneJET Genomic DNA Purification Kit (Thermo Fisher Scientific, Waltham, MA, USA). The *16S rRNA* gene sequence was amplified using the universal primers E8F (5′-AGAGTTTGATCCTGGCTCAG-3′) and E1541R (5′-AAGGAGGTGATCCAGCC-3′) [27]. The PCR reaction (25 μL volume) contained 12.5 μL of TaqMan Environmental Master Mix Taq (Thermo Fisher Scientific), 0.5 μM primer, and 1 μL of DNA template and was performed under the following conditions: initial denaturation at 94 °C for 2 min, 30 cycles of 94 °C for 30 s, 55 °C for 30 s, and 72 °C for 1 min, followed by a final elongation at 72 °C for 10 min. The amplified products were purified with MagMax magnetic beads (Thermo Fisher Scientific) and sequenced from both ends using the same primers (BaseClear, Leiden, The Netherlands). The NCBI Core nucleotide database [28] was queried on the NCBI BLASTN server v.2.16.1+ [29] using identity cut-off values of 98.65% for species [30] and 95% for genus [31].

### 2.3. Metagenomic Analysis of the Rhizome Microbial Communities

For DNA extraction, three independent plant replicates were selected for each experimental group of the 1-year-old and >4-year-old plants that are indicated in the text and figures as samples A1-3 and B1-3, respectively. One to four DNA samples were prepared for each plant replicate. Total genomic DNA was extracted from 0.2 g of the sterilised rhizome samples by using the modified cetyltrimethylammonium bromide method [32], with the incubating time at 65 °C prolonged to 60 min. The quantity and quality of the isolated genomic DNA were evaluated using a micro-volume spectrophotometer, and accurate quantification of dsDNA was performed with Qubit 2.0 fluorometer and Qubit dsDNA Quantification Assay Kits (Thermo Fisher Scientific) according to the manufacturer’s protocol. After extraction, the DNA samples were stored and frozen at −20 °C until being sent on dry ice for metagenomic analysis. Amplicon DNA fragment library preparation and sequencing of the eluted DNA samples were performed at BMKGENE (Münster, Germany) using the Illumina NovaSeq system (Illumina, San Diego, CA, USA). Variable domain 4 (V4) of the *16S rRNA* gene fragment was amplified using 515F 5′-GTGYCAGCMGCCGCGGTAA-3′ [33] and 806R 5′-GGACTACNVGGGTWTCTAAT-3′ [34] primers, and variable domains 5–7 (V5–7) were amplified using 799F 5′-AACMGGATTAGATACCCKG-3′ [35] and 5′-ACGTCATCCCCACCTTCC-3′ [36] primers. Sequence data were processed using R v.4.2.1 [37]. Primer sequences were removed using Cutadapt v.5.0 [38]. Reads were filtered and trimmed using the *DADA2* v.1.8 [39] pipeline and the following parameters: truncLen = c(220,210), maxN = 0, maxEE = c(2,2), and truncQ = 2. Reads were merged, and the resulting ASVs were further processed to remove chimeras. Taxonomy was assigned using *DADA2* naïve Bayesian classifier and Silva version 138.2 database [40].

### 2.4. Assessment of the Functional Genes

Functional gene amplification was performed using primers and PCR conditions previously described for nitrogenase *nifH* [41], nitrate reductase *narG* [42], alkaline phosphatase *phoD* [43], phosphonatase *phnX* [44], quinoprotein glucose dehydrogenase *gcd* [45], and pyrroloquinoline-quinone synthase *pqqC* [46] genes. PCR products were analysed using the MultiNA microchip electrophoresis system (Shimadzu, Kyoto, Japan), and the presence of the amplicons of previously published size was considered as a positive result.

### 2.5. Biochemical Analysis of Plant Growth-Promoting Traits

Nitrogen fixation was screened on a semisolid nitrogen-free medium [47]. Siderophore production activity was detected by growth on the chrome azurol S medium prepared as described by Schwyn and Neilands [48]. The ability to solubilise tricalcium phosphate was tested on a modified Pikovskaya medium as described by Edi-Premono et al. [49]. To estimate the synthesis of indole-3-acetic acid and other indole-related compounds (IRCs), bacteria were cultured in yeast extract mannitol broth supplemented with 500 μg mL^−1^ L-tryptophan as described by Husen [50], and IRCs were quantitated calorimetrically using the ferric chloride perchloric acid reagent [51].

### 2.6. Assessment of Intracellular Redox Modulation by Bacterial Isolates Using Tobacco Cells

*Nicotiana tabacum* L. cv. Samsun-NN cell suspension and bacteria isolate co-cultivation were performed as described by Tamošiūnė et al. [52]. Plant cells and bacteria were used at the exponential growth phase. Bacteria were sedimented by centrifugation, resuspended in Murashige–Skoog medium [53], and used to inoculate plant cells at a final density of 0.1 OD. After 6 h incubation, intracellular redox balance was estimated using dichlorofluorescein diacetate staining and fluorometer LS55 (Perkin-Elmer, Waltham, MA, USA), as described by Joo et al. [54].

### 2.7. Arabidopsis Seedling Growth-Modulating Activity of Bacterial Isolates

*Arabidopsis thaliana* L. seeds were sown on filter paper in a Petri dish and soaked with 1 ml of deionised water. The plates were stored at 4 °C for 48 h. Bacterial isolates cultured in liquid LB broth overnight were centrifuged, resuspended in deionised water at 0.1 OD mL^−1^, and used for seed inoculation at 1 mL per Petri dish. Deionised water without bacteria was used for the control treatment. Plates were incubated in the growth chamber at 25 ± 3 °C under fluorescent lamp illumination at 150 µmol m^−2^ s^−1^ intensity with a 16/8 h light/dark photoperiod for 48 h. To visualise root growth, seedlings were stained with 1 mg mL^−1^ nitro blue tetrazolium solubilised in 50 mM NaPO_4_, 0.02% NaN_3_, and fixed with ethanol/glycerol/acetic acid (3:1:1). Images were captured using a Nikon SMZ1000 microscope (Nikon, Tokyo, Japan), and seedling radicle and hypocotyl length were measured using ImageJ software v. 1.54 [55].

### 2.8. Data Analysis

A phylogenetic relationship of the bacterial isolates was visualised using the Interactive Tree of Life (iTOL) server v.7 [56] and the identified representative sequence data.

For the metataxonomic analysis, the data of DNA extraction and sequencing repeats were combined for each plant replicate. Statistical data analysis was performed using the Marker Data Profiling function of the MicrobiomeAnalyst server v.2.0 [57]. ASVs with <10 counts and <10% prevalence in the sample were removed, data were rarefied to the minimum library size of 2538 reads, and total sum normalisation was applied by dividing feature read counts by the total number of reads in each sample. The Bray–Curtis dissimilarity matrix was applied in nonmetric multidimensional scaling (NMDS) analysis. The threshold of >5% counts and >30% samples was used for the core microbiome calculation. R package *Durga* v.2.0 [58] was used to assess mean differences of alpha-diversity indices.

Hierarchical cluster analysis of taxonomic, genetic, and biochemical bacterial isolate data was performed using the *daisy* function and Gower distance matrix of the R package *cluster* v.2.1.8 [59]. Genetic traits were treated as asymmetric binary variables to minimise a bias of the false negative results associated with primer taxonomic specificity.

Statistically significant differences between the means of the Arabidopsis seedling measurements were assessed by ANOVA analysis and Tukey’s post hoc test using R package *agricolae* v.1.3-7 [60] and visualised by *gglot2* v.3.5.1 package [61].

## 3. Results

### 3.1. Diversity of Endophytic Bacteria in R. rosea Rhizome

**Figure 1:**
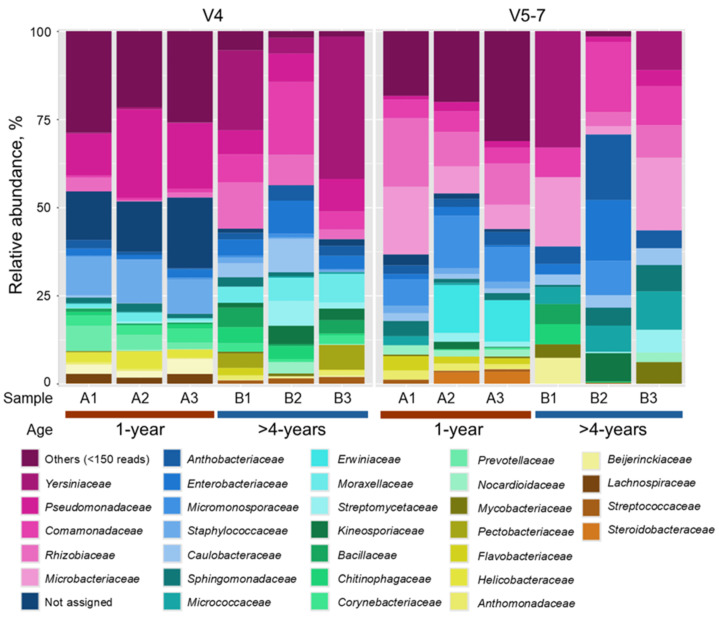
Composition and relative abundance of family-level endophytic bacterial taxa in 1-year-old and >4-year-old *R. rosea* rhizome samples assessed using metataxonomic sequencing of *16S rRNA* variable region V4 and V5–7 amplicons.

Metataxonomic *16S rRNR* gene domain V4 and V5–7 high-throughput sequencing analysis was performed to assess bacterial diversity in the *R. rosea* rhizome and its variation depending on plant age. Three plant replicates were used for each of the two experimental groups of 1-year-old and >4-year-old plants (Supplementary Materials, Table S1). The analysis yielded the total number of mapped reads from 108,891 to 328,371 and from 241,393 to 589,600 for domains V4 and V5–7, respectively. However, the proportion of reads mapped to bacterial amplicon sequence variants (ASVs) varied depending on the sample and *16S rRNR* gene domain (Supplementary Materials, Figure S1). For domain V4, the proportion of bacterial reads varied from 1 to 24%. A higher proportion of bacterial reads was obtained using V5–7 specific primers, ranging from 6 to 91%. Differences in bacteria abundance in the rhizome samples could be implied from the variation in bacterial read proportion since the latter depends on the ratio of bacterial DNA to plastid and mitochondrial DNA content in the sample [62]. Overall, 4148–57,608 and 7360–218,571 bacterial reads obtained for domains V4 and V5–7 were assigned to 108 bacterial families (Figure 1; Supplementary Materials, Table S2).

Primers specific to different variable domains of the *16S rRNA* gene often have different taxonomic coverage. Domain V4 amplicons are often used for metataxonomic analysis and provide broad taxonomic coverage [63,64]. However, in our study, several families of Actinobacteria (e.g., *Microbacteriaceae*, *Micromonosporaceae*, *Micrococcaceae*, and *Cellulomonadaceae*) were largely under-represented in the V4 dataset compared to the results obtained using amplicons corresponding to the domains V5–7. Conversely, the use of domain V5–7-specific primers resulted in less efficient or no detection of certain Gammaproteobacteria, including order *Enterobacterales* (e.g., *Yersiniaceae*, *Pectobacteriaceae*), *Pseudomonadales* (*Moraxellaceae*, *Pseudomonadaceae*), or Actinobacteria (e.g., *Corynebacteriaceae*). Therefore, combined data of the two *16S rRNA* gene regions were used for bacterial diversity analysis.

**Figure 2:**
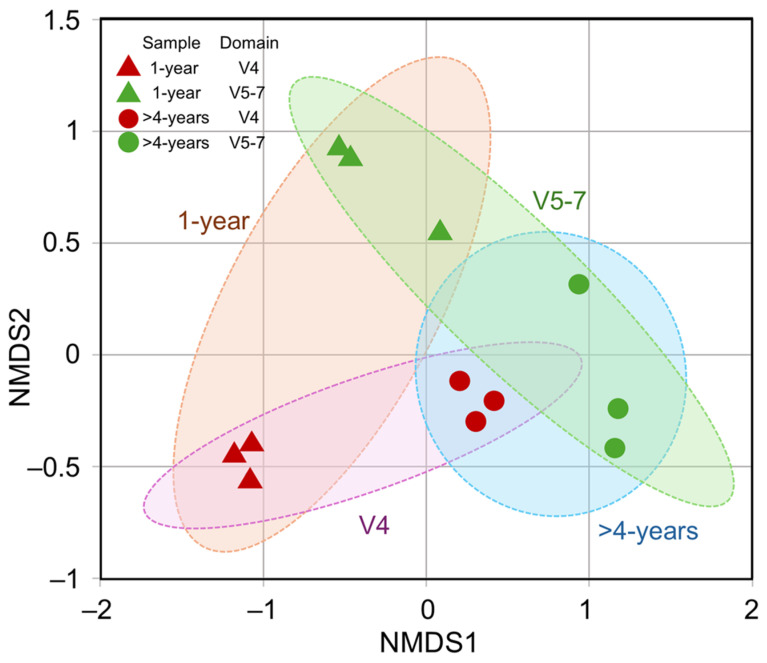
Bacterial diversity variation in 1-year-old and >4-year-old *R. rosea* rhizome samples. The nonmetric multidimensional scaling (NMDS) analysis of the ASV datasets generated using metataxonomic sequencing of *16S rRNA* variable region V4 and V5–7 amplicons was carried out using the Bray–Curtis dissimilarity matrix.

The NMDS analysis revealed a significant (*p* < 0.005) difference in bacterial population composition between 1-year-old and >4-year-old plant rhizome samples. This effect was corroborated by the results obtained from both *16S rRNA* gene amplicon datasets (Figure 2). The differences between the two experimental groups were related to the variation in bacterial diversity (richness of taxa and evenness of abundance distribution) as well as distinct taxonomic composition. Based on the Observed or Chao1 diversity index, the rhizome of 1-year-old plants had approximately three-fold higher richness of bacterial taxa (Table 1; Supplementary Materials, Figure S3). Although a large effect size was estimated for all indices, the differences were less prominent for Shannon, Simpson, or Fisher’s alpha indices, which consider the evenness of taxa distribution within the microbial community. The results suggest that certain taxa tend to dominate in young plant rhizomes, and the bacterial diversity is reduced in >4-year-old plant rhizome samples, but the relative abundance of taxa is more evenly distributed.

**Figure 3:**
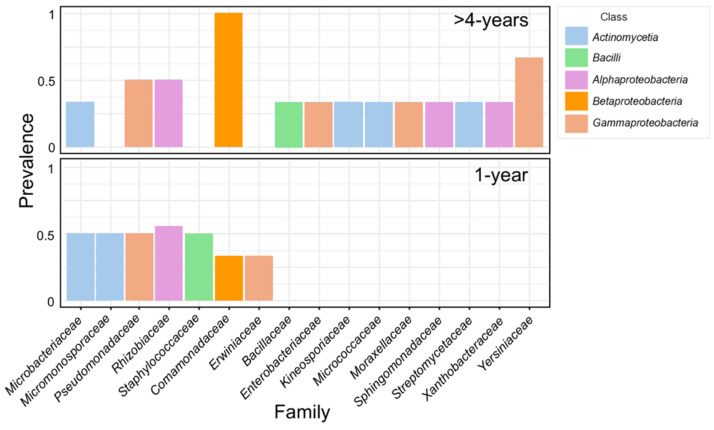
Core microbiome of *R. rosea* 1-year-old (**upper panel**) and >4-year-old (**lower panel**) rhizome samples estimated based on *16S rRNA* variable region V4 and V5–7 amplicon metataxonomic sequencing. The threshold of >5% counts and >30% samples was used for calculation using the MicrobiomeAnalyst server [57]. Bacterial classes are represented by different colours.

*Staphylococcaceae* and Pseudomonadaceae were identified as dominant bacterial families in the 1-year-old plant rhizome using domain V4-specific primers. In addition, analysis of the V5–7 amplicon dataset revealed a predominance of *Rhizobiaceae*, *Microbacteriaceae*, and *Micromonosporaceae* (Figure 1; Supplementary Materials, Table S2). Conversely, *Yersiniaceae* and *Comamonadaceae* were detected as the dominant families in the >4-year-old plant rhizome samples using both primer sets. The core microbiome of the two experimental groups shared three families of Proteobacteria (*Comamonadaceae*, *Pseudomonadaceae*, and *Rhizobiaceae*) and Actinobacteria of the *Microbacteriaceae* family; meanwhile, another three and nine families of Proteobacteria, Firmicutes, and Actinobacteria were unique to the 1-year-old and >4-year-old plant rhizome samples, respectively, as shown in Figure 3. It is notable that ASVs representing families such as *Xanthobacteriaceae*, *Erwiniaceae*, and *Pectobacteriaceae* that include a number of pathogenic species were detected in significant abundance (up to 7–18% of reads) either in the core microbiome or individual plant samples.

Alpha diversity analysis was performed using the Microbiome Analyst server [57], and the statistical significance of the differences between the means of the two experimental groups was assessed using the R package *Durga* v.2.0 [58]. Data are presented as the mean and standard deviation. Hedge’s g standardised mean difference effect size estimates were calculated with a 95% confidence interval (95% CI).

### 3.2. Diversity of Culturable Endophytic Bacteria

**Figure 4:**
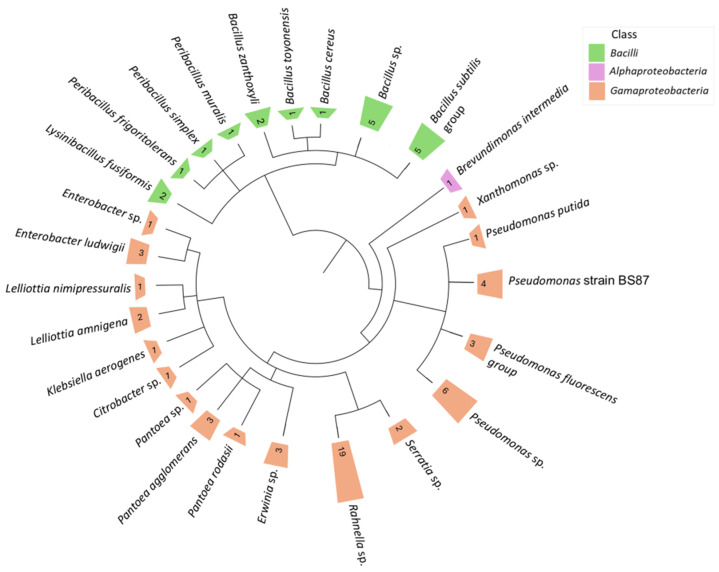
Phylogenetic tree reconstruction for endophytic isolates obtained from the rhizome of *R. rosea*. A phylogenetic tree was built using the iTOL server v.7 [56] and representative sequence data. The numbers represent the count of isolates.

In total, 73 putative endophytic bacterial isolates were obtained from healthy *R. rosea* rhizomes of 1-year-old and >4-year-old plants. Although a larger number of isolates was obtained from the rhizomes of mature plants (54 isolates) compared to the rhizomes of 1-year-old plants (19 isolates), the composition of the isolates was similar for both sources (Supplementary Materials, Table S3). Based on *16S rRNA* gene sequence and phylogenetic tree reconstruction using representative taxon sequences (exhibiting 98–100% homology), the isolates were assigned to the phyla of Firmicutes (26%) and Proteobacteria (74%) (Figure 4). Three genera of Firmicutes included *Peribacillus* (4.1%), *Lysinibacillus* (2.7%), and *Bacillus* (19.2%). The majority (74%) of Proteobacteria isolates belonged to two different classes: *Gammaproteobacteria* and *Alphaproteobacteria*. *Gamaproteobacteria* were mostly isolated from rhizome of mature plants and were represented by ten genera, including *Rahnella* (26%), *Pseudomonas* (19.2%), *Pantoea* (6.8%), *Enterobacter* (5.5%), *Erwinia* (4.1%), *Lelliottia* (4.1%), *Serratia* (2.7%), *Xanthomonas* (1.4%), *Klebsiella* (1.4%), and *Citrobacter* (1.4%). On the contrary, the only isolate assigned to *Alphaproteobacteria* was isolated from young plant rhizome and belonged to the genus *Brevundimonas*.

### 3.3. Genetic and Biochemical Plant Growth-Promoting Traits of the Bacterial Isolates

All bacterial isolates were screened by PCR for functional genes related to nitrogen fixation (*nifH*), nitrate reduction (*narG*), organic phosphorus mineralisation (*phoD* and *phnX*), and inorganic phosphorus dissolution (*gcd* and *pqqC*). Although PCR-based microbial metabolic gene analysis is often limited by primer taxonomic specificity and only partially uncovers metabolic diversity [65], the obtained information still provides a significant contribution to bacterial isolate classification according to the metabolic traits. As it is important to avoid a bias of false negative detection results, the gene screening data were treated as asymmetric binary variables in the cluster analysis, thus considering only positive detection results in the assignment of clusters. Further, nitrogen fixation and inorganic phosphorus solubilisation traits were analysed using biochemical assays.

**Figure 5:**
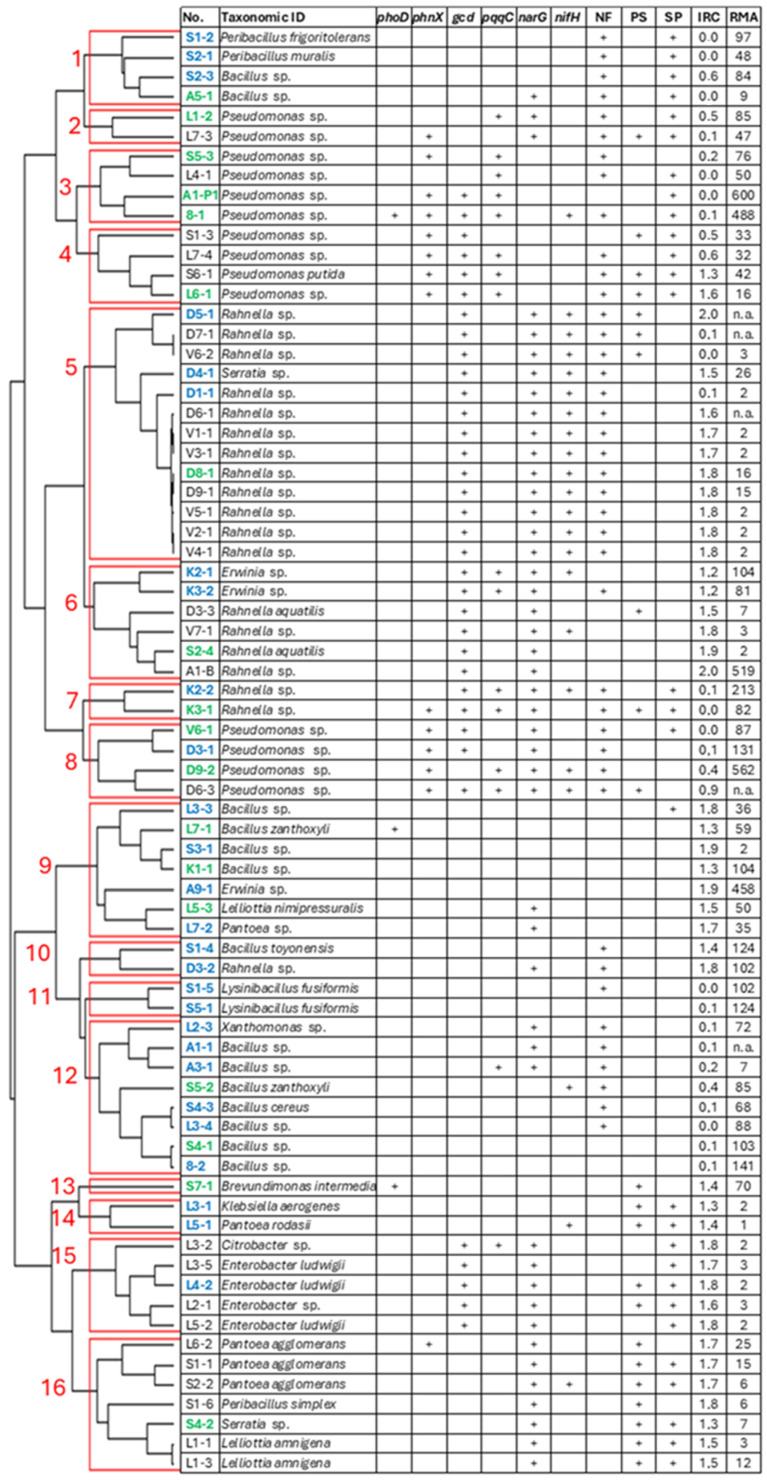
Plant growth-promoting traits of endophytic bacteria isolated from the rhizome of *R. rosea*. The numbers in red font indicate representative clusters. The isolate numbers shown in blue or green colour font indicate isolates selected for growth regulation analysis using Arabidopsis seedlings, and those marked in green colour font showed the most prominent growth-enhancing effect. Positive test results are indicated by “+” sign. Abbreviations: NF—nitrogen fixation; PS—phosphate solubilisation; SP—siderophore production; IRC—indole-related compound production (mg mL^−1^); RMA—cellular redox-modulating activity in tobacco cell culture (% of control).

Fifty-eight isolates yielded at least one positive result of the functional gene screening assay (Figure 5). Among the genes used in the analysis, alkaline phosphate *phoD* was the least common among the isolates and was detected only for three isolates of *Pseudomonas*, *Bacillus*, and *Brevundimonas* spp. The phosphonatase-encoding *phnX* gene was detected for the majority of Pseudomonads of clusters 2–4 and 8 that corresponded to 19% of all isolates. These isolates were also mostly positive for glucose dehydrogenase and pyrroloquinoline quinone synthase genes involved in the production of gluconic acid and, thus, inorganic phosphorus solubilisation. However, only approximately half of these isolates could solubilise tricalcium phosphate in the in vitro assay, suggesting variation in levels of the *gcd* and/or *pqqC* expression among the isolates under the in vitro assay conditions. Several bacteria assigned to clusters 13–16 showed tricalcium phosphate solubilisation activity that is likely related to the production of organic or inorganic acids other than gluconic acid. Overall, 30% of isolates were able to dissolve phosphates in the in vitro assay.

More than half (56%) of the isolates were able to grow in the absence of a nitrogen source in the medium. For the majority of *Rahnella* sp. isolates assigned to cluster 5, and also three *Pseudomonas* sp. isolates (D6-3, D9-2, and 8-1) and *Bacillus zanthoxyli* S5-2, the presence of *nifH* was associated with positive results of the nitrogen fixation assay. Gene *nifH* was detected in 30% of isolates; however, another 26% of isolates that included the majority of clusters 1–4, 7–8, and 10–12, mostly assigned to *Bacillus*, *Peribacillus*, and *Pseudomonas* spp., showed detectable growth on the medium without a nitrogen source. Thus, it could be suggested that, for these bacteria, the *nifH* gene sequence variation rendered it undetectable in the PCR assay. The nitrate reductase gene *narG* was rather common (63%) among the isolates, and it was not detectable only among several *Bacillus*, *Peribacillus*, and *Pseudomonas* sp. assigned to clusters 1–4 and 9–14.

Siderophore production was mostly limited to clusters 1–4, 7, and 15–16 that altogether constituted 40% of all isolates and were assigned mostly to *Pseudomonas* sp. but also *Bacillus*, *Peribacillus*, *Enterobacter*, *Pantoea* spp., or other genera. Similarly, the production of indole-3-acetic acid or other IRCs was detectable for more than half (51%) of the isolates and varied from 0.1 to 2 mg mL^−1^. Under in vitro conditions, the highest producers of IRCs were *Rahnela* sp. isolates A1-8 and D5-4 (2 mg mL^−1^); however, overall, 40% of the isolates showed IRC production higher than 1.5 mg mL^−1^.

Most endophytic bacteria are capable of either avoiding or suppressing the microbe-associated pattern-triggered response of the host plant. To assess bacterial isolates’ capacity to engage in the endophytic interaction with plant cells, bacterial isolates were co-cultivated with tobacco plant cell suspension culture, and cell redox balance-modulating activity was assessed [52]. Approximately 25% of isolates induced low or moderate changes in the cell’s redox balance compared to control cells. This effect was prevalent among the bacteria of clusters 1–4, 7–8, and 10–12, and it implied that the isolates did not trigger or suppress immediate defence response and would likely be able to establish compatible interaction with the plant cells. Contrarily, half of the isolates had strong reactive oxygen species production-suppressing activity, which was likely a consequence of a strong defence response leading to the death of the tobacco cells. However, one must consider that a microbe-triggered defence response is often host-plant specific; therefore, the observed results might vary for different plant species.

### 3.4. Plant Growth-Modulating Activity of the Bacterial Isolates

**Figure 6:**
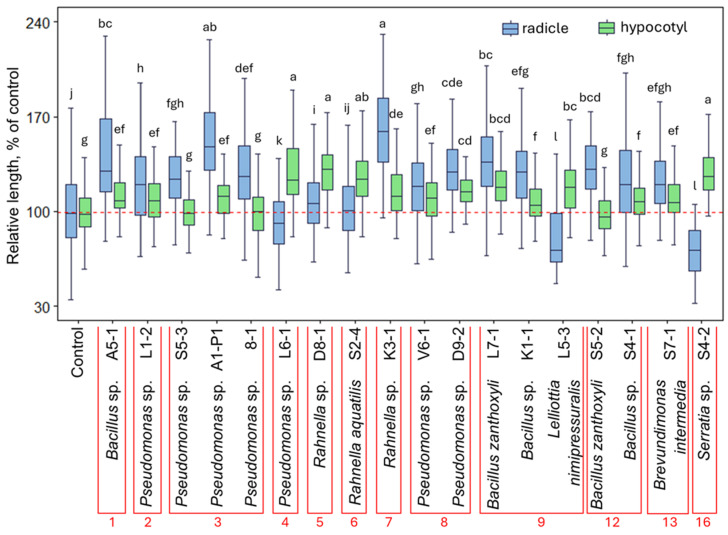
Arabidopsis seedling radicle (blue fill colour)- and hypocotyl (green fill colour)-growth-modulating effect of selected endophytic bacterial isolates obtained from *R. rosea* rhizome. The data are shown as boxplots representing the median, minimum and maximum scores, and lower and upper quartiles; the red dashed line indicates the mean value of control; numbers indicate clusters as shown in Figure 5; different letters denote significant differences between the analysed groups (*p* ≤ 0.05).

To further assess the plant growth-promoting capacity of the *R. rosea* endophytes, 45 isolates were selected based on the results of genetic and biochemical plant growth-stimulating trait analysis. The bacteria were co-cultivated with germinating Arabidopsis seedlings, and radicle and hypocotyl growth-modulating effects were assayed. Similarly to previously described co-cultivation experiments [52,62,66], bacteria were inoculated at an OD of 0.1, which corresponds to a cell density of approximately 5 × 10^7^ to 1.5 × 10^8^ cells mL^−1^ estimated for related Firmicutes and Proteobacteria such as *Bacillus*, *Pseudomonas*, or *Escherichia* spp. [67]. A significant radicle- and hypocotyl-growth-stimulating effect was detected for 23 and 27 isolates, respectively (Supplementary Materials, Figures S4–S6). Root growth response to the bacteria co-cultivation was more prominent, and approximately half (13) of the selected isolates stimulated root growth by more than 20%, including *Rahnella* sp. K3-1 and *Pseudomonas* sp. A1-P1, which resulted in 62 ± 4% and 52 ± 6% increased radicle length compared to the control, respectively (Figure 6). Conversely, the bacteria co-cultivation effect on seedling shoots was less pronounced, and only five isolates stimulated hypocotyl growth by more than 20%. *Pseudomonas* sp. L6-1 and *Rahnella* sp. D8-1 had the strongest effect, resulting in 30 ± 3% and 30 ± 2% increases in hypocotyl length compared to the control, respectively. Although several isolates had significant growth-promoting effects on both the radicle and hypocotyl, the effect was always stronger (larger than 20%) for one of the seedling parts. It is also notable that several bacteria suppressed seedling growth. The most prominent effect was observed for *Xanthomonas* sp. L2-3, which resulted in 52 ± 2% and 13 ± 1.3% reduced radicle and hypocotyl length compared to control, respectively. Therefore, the presence of the bacterium in *R. rosea* rhizobium is rather a consequence of pathogenic infection than endophytic colonisation.

## 4. Discussion

The structure of plant-associated microbial communities is defined by complex interactions between hosts, microbes, and associated environmental factors such as soil, climate, or agricultural practices [68,69]. Plant ageing-associated physiological and molecular processes, such as changes in root structure and secretion of root-derived exudates, represent one of the factors that affect the recruitment and enrichment of microbiota in the rhizosphere and root endosphere, which leads to a succession of plant-associated microbiota over time [70,71]. Previous studies have described the changes in microbiome richness and composition over the lifetime of perennial plants, including *Boechera stricta* (*Brassicaceae*) [71], *Arabis alpina* [72], and ligneous plants, such as grapevine [73,74,75] or trees of *Populus* sp. [76].

In our study, the results of metataxonomic analysis revealed age-related changes in bacterial diversity and composition in the rhizome of *R. rosea* plants (Figure 1 and Figure 3). Young plants exhibited a high abundance of *Pseudomonadaceae* and *Staphylococcaceae* which could be related to the vertical transmission of endophytic bacteria through seeds, leading to seedling colonisation. The two bacterial families include endophytic species previously identified in various plant seeds [77]. Microbial composition changes during rhizome development might also be associated with growth-related structural and metabolic transformations, along with extrinsic factors like an increased incidence of pathogen infection. Indeed, bacteria of the *Xanthobacteraceae* and *Pectobacteriaceae* families that include known plant pathogens were detected in the rhizome of the mature (>4-year-old) *R. rosea* plants (Figure 1; Supplementary Materials, Table S2). Several species of *Pectobacteriaceae* are aggressive plant root necrotrophic pathogens that harbour a large arsenal of plant-cell-wall-degrading enzymes and affect a broad range of plant hosts [78]. *Xanthobacteraceae* includes several species of well-studied bacterial plant pathogens that cause mostly leaf and fruit diseases in a variety of plants [79]. In our study, the *Xanthomonas* sp. L2-3 isolate suppressed the root growth of Arabidopsis seedlings (Supplementary Materials, Figure S5), indicating that the bacterium could potentially negatively impact the roots of *R. rosea*. Notably, isolates of *Erwinia* sp. (K3-1, K3-2, and A9-1) had no adverse effect on Arabidopsis seedling growth (Supplementary Materials, Figures S4 and S5). This would suggest that ASVs of *Erwiniaceae* family detected in the rhizome of 1-year-old plants using metataxonomic analysis may not be related to pathogenic species. However, further analysis of the ecological significance and potential pathogen-suppressing activity of endophytic bacteria inhabiting *R. rosea* rhizome would be of interest for future studies.

Previous studies with Arabidopsis [80] and grapevine [81] revealed that plant infection by pathogens led to increased species richness of beneficial microbiota in the rhizosphere. It was established that disease or environmental stress can promote the recruitment of beneficial microorganisms by upregulating exudate production [82,83]. Such phenomena would explain the increased abundance of several families of Proteobacteria, Firmicutes, and Actinobacteria in the mature *R. rosea* plant rhizome, as observed in our study (Figure 1; Supplementary Materials, Table S2). In particular, this includes bacteria of the *Yersiniaceae* family that were found predominant in the rhizome of mature plants and could potentially play a growth-promoting or antagonistic role in the response to pathogen infection. Interestingly, a majority of the isolates of *Rahnella* spp., representing the *Yersiniaceae* family, were obtained from mature plant rhizome samples (Supplementary Materials, Table S3), and the plant growth-promoting properties of several isolates were confirmed by metabolic tests and the Arabidopsis seedling co-cultivation assay (Figure 5 and Figure 6; Supplementary Materials, Figures S4 and S5).

Genus *Rahnella* comprises thirteen species of *Gammaproteobacteria* that have been isolated from a broad range of environments, such as soil, water, seed, food, plants, insects, and clinical samples [84,85]. The genus includes many symbionts and harmless plant-associated bacteria with the potential to protect plants from pathogens and benefit plant growth [86,87,88]. In addition, several studies have revealed that plant colonisation by bacteria of *Rahnella* spp. is associated with extreme environments [85,89,90]. For example, the novel *Rahnella sikkimica* sp. nov. was described as a bacterial strain isolated from an extremely cold environment that possesses plant growth-promoting properties [89,90]. In our study, two isolates of *Rahnella* spp. were assigned to *R. aquatilis* species. Plant growth-promoting properties of this species were the focus of several previous studies. *R. aquatilis* strain JZ-GX1 increased the contents of chlorophyll, active iron, and the activities of antioxidant enzymes in leaves of *Cinnamomum camphora* [91]. It was further established that the bacterium could benefit plant growth through various mechanisms, such as nitrogen fixation, phytohormone production, phosphate solubilisation through biosynthesis of organic acids and pyrroloquinoline quinone [87], production of antibacterial substances [92], secretion of volatile organic compounds [93], and siderophore-producing activity [94]. Our study suggests that the nitrogen fixation or indole-related compound-producing activity of the *Rahnella* sp. isolates may contribute to the positive effect on Arabidopsis seedling root and shoot growth (Figure 5 and Figure 6).

Several *Pseudomonas* sp. isolates that showed plant growth-enhancing properties in our study (Figure 5 and Figure 6) belong to another family of *Gammaproteobacteria*. *Pseudomonas* is one of the most abundant genera of beneficial rhizobacteria, and their endophytic, plant growth-promoting, and antagonistic properties have long been established [95]. Plant-associated *Pseudomonas* spp. can produce secondary metabolites such as indole acetic acid and siderophores and can also solubilise phosphate [96]; therefore, many strains of Pseudomonads attracted attention as a source of bacterial fertilisers or biological control agents [97].

Firmicutes is also one of the richest phyla of plant growth-promoting bacteria. In the current study, several isolates of *Bacillaceae* showed nitrogen fixation, indole-related compounds, and siderophore-producing activity, as well as stimulated the growth of Arabidopsis seedlings (Figure 5 and Figure 6), which could be related to nitrogen fixation or indole-related compound-producing activity. Plant growth-enhancing compound-producing capacity was established for numerous species belonging to the genus *Bacillus* [98]. Among the identified species, *Bacillus zanthoxyli* was previously reported as having a prominent effect on enhancing plant resistance against various stresses [99]. For instance, it significantly increased the number of tillers and leaf width of tall fescue seedlings under salt conditions [100]. Additionally, it improved the synthesis of photosynthetic pigments, which led to improved growth of cabbage, tomato, and cucumber [99]. Although members of the *Bacillaceae* family have been widely used in agriculture, *Lysinibacillus* and *Peribacillus* spp. are also considered as plant growth boosters. Recently, Marik et al. [101] have shown that *P. frigoritolerans* is a potential biofertiliser that regulates plant genes to promote growth and drought tolerance. In addition, *Lysinibacillus fusiformis* Lf89 was shown to stimulate the root growth and proliferation of Arabidopsis and Datura plants [102,103].

Bacteria of *Bacillus* and *Pseudomonas* sp. are the most widely used in the agro-biotech industry as phytostimulants and biofertilisers due to the variety of plant growth-promoting activities and the ability to maintain viability in biofertiliser formulations over long time periods [104,105]. Although *Rahnella* sp. has, so far, remained in the shadow of its more successful competitors, the development of mineral-solubilising bioformulations including a strain of *R. aquatilis* has been described recently [106]. Therefore, seven *Pseudomonas* sp., five *Bacillus* sp., and three *Rahnella* sp. isolates obtained from the *R. rosea* rhizome that possess significant plant growth-stimulating activity (Figure 6) represent a promising target for future genetic and metabolic studies which could potentially reveal novel bacterial strains suitable for biofertiliser production.

Cultivation-based methods provide means to directly study bacterial genetics and metabolic activity; however, many microbial species with important ecological functions in the environment and plant microbiome could not be grown under laboratory conditions. Thus, DNA sequence analysis-based techniques proved to be an effective strategy for uncovering the uncultivable part of microbial communities [107]. Likewise, in our study, no cultivable isolates were obtained for *Comamonadaceae*, *Rhizobiacea,* or several Actinobacteria families that constituted a part of the core microbiome of the mature plant rhizome detected using metataxonomic analysis (Figure 3 and Figure 4). Although plant growth-promoting properties of these bacteria could not be verified, it is notable that these taxa include numerous species of beneficial plant microbiota. Soil-thriving bacteria of the *Burkholderia* genus, which is a member of the *Comamonadaceae* family, have been reported to colonise more than 30 plant species [108]. It has been shown that bacteria of the *Rhizobiaceae* family can associate with non-legume roots, which ultimately leads to the stimulation of growth through phytohormone production, phosphate solubilisation, or the production of siderophores [109]. *Micromonosporaceae* includes endophytic actinobacteria species possessing growth-promoting and antifungal properties [110].

## 5. Conclusions

Our study provides new insights into the complexity of the endophytic bacteria community of the succulent plant *R. rosea* rhizome and the succession of microbial composition related to plant ageing. *Staphylococcaceae*, *Pseudomonadaceae*, *Rhizobiaceae*, *Microbacteriaceae*, and *Micromonosporaceae* dominated in the rhizome of young *R. rosea* plants among the 108 bacterial families detected by *16S rRNA* gene amplicon metataxonomic analysis. Though a significant decrease in microbial diversity is observed as the plant ages, *Yersiniaceae* and *Comamonadaceae* predominate in the core microbiome of the mature plant rhizome. The reshaping of the microbial community could be partially linked to the mature plant rhizome disposition to the infection by bacterial pathogens that could also lead to the recruitment of beneficial microbiota. As our study was limited to plant-age-related changes in the endophytic bacteria community, further investigation of the effect of environmental factors, such as soil, climatic, and cultivation conditions, on the diversity and dynamics of the plant-associated microbiota would be required to fully uncover the potential of the succulent plant microbiome.

A cultivation-based approach led to the isolation of *R. rosea* rhizome endophytic bacteria that were assigned to 14 distinct bacterial genera, many of which possessed genetic or biochemical traits essential for plant nutrient uptake and growth enhancement and exhibited potential for compatible endophytic interaction. Many of the cultivable bacteria isolates represented well-known and extensively studied plant endophytic and plant growth-promoting groups of bacteria, including Pseudomonads and several genera of Bacilli. In addition, the *Rahnella* genus, a representative of the *Yersiniaceae* family, was also prominent among the isolates, suggesting its significant role in the *R. rosea* rhizome microbial community that could potentially be involved in growth-promoting or antagonistic functions. This study identified several bacterial isolates with plant growth-promoting properties that present a promising target for consideration in the design of phytostimulant formulations suitable for sustainable and environment-friendly agriculture practices.

## Figures and Tables

**Table 1:** Alpha diversity indices of *R. rosea* rhizome samples estimated using *16S rRNA* domain V4 and V5–7 amplicon sequencing analysis.

Index	1-Year	>4-Year	*p*-Value	Hedge’s g	95% CI
V4					
Observed	83.7 ± 7.2	29.7 ± 1.5	0.004	6.25	5.56, 25.09
Chao1	84.0 ± 7.0	29.7 ± 1.5	0.004	6.51	5.56, 32.56
Shannon	3.5 ± 0.2	2.6 ± 0.2	0.008	3.21	2.05, 5.05
Simpson	0.93 ± 0.02	0.87 ± 0.06	0.189	0.98	0.50, 1.59
Fisher’s alpha	16.7 ± 1.8	4.7 ± 0.3	0.007	5.43	4.94, 6.20
V5–7					
Observed	54.0 ± 16.5	13.0 ± 1.7	0.048	2.01	1.41, 25.59
Chao1	54.1 ± 16.5	13.0 ± 1.7	0.048	2.01	1.41, 53.99
Shannon	3.2 ± 0.3	2.2 ± 0.1	0.013	3.16	1.81, 10.14
Simpson	0.93 ± 0.02	0.86 ± 0.03	0.053	1.80	0.75, 3.47
Fisher’s alpha	9.8 ± 3.5	1.8 ± 0.3	0.058	1.83	1.27, 32.69

## Data Availability

The original contributions presented in this study are included in the article/Supplementary Material. Further inquiries can be directed to the corresponding author.

## Supplementary Materials

The following supporting information can be downloaded at https://www.mdpi.com/article/10.3390/microorganisms13010013/s1: Figure S1: Rarefaction curves for *R. rosea* rhizome samples estimated from *16S rRNA* gene high-throughput sequencing analysis data using the Microbiome Analyst server (Chong et al., 2020 [57]); Figure S2: Distribution of the bacterial (red) to plastidial (green) and mitochondrial (blue) reads obtained from *16S rRNA* gene high-throughput sequencing analysis datasets of 1-year-old and >4-year-old *R. rosea* rhizome samples; Figure S3. Comparison of alpha diversity indices of *R. rosea* rhizome samples estimated using 16S rRNA domain V4 (upper panels) and V5–7 (lower panels) amplicon sequencing analysis. Figure S4. Representative images of Arabidopsis seedling shoot growth-modulating effect of endophytic bacterial isolates obtained from *R. rosea* rhizome; Figure S5: Arabidopsis seedling root growth-modulating effect of endophytic bacterial isolates obtained from *R. rosea* rhizome; Figure S6: Arabidopsis seedling shoot growth-modulating effect of endophytic bacterial isolates obtained from *R. rosea* rhizome; Table S1: Summary statistics *16S rRNA* gene metataxonomic analysis of the *R. rosea* rhizome samples; Table S2: Relative abundance of family-level taxa in *Rhodiola rosea* plant rhizome samples; Table S3: Results of bacterial isolate identification using *16S rRNA* gene sequence data.
