# Supplementary material for: Diversity and Plant Growth-Promoting Properties of Rhodiola rosea Root Endophytic Bacteria
Source: Microorganisms. 2024 Dec 25;13(1):13. doi: 10.3390/microorganisms13010013 (PMC11767865; doi:10.3390/microorganisms13010013)
Supplement: Supplementary file 1 [file microorganisms-13-00013-s001.zip › Supplementary-Materials.pdf]

Supplementary Materials

Diversity and plant growth-promoting properties of *Rhodiola rosea* root endophytic bacteria

Inga Tamošiūnė [1], Muhammad Fahad Hakim [1], Odeta Buzaitė [2], Vidmantas Stanys [1], Jurgita Vinskienė [1], Elena Andriūnaitė [1], Danas Baniulis [1,*]

[1] Institute of Horticulture, Lithuanian Research Centre for Agriculture and Forestry, Kaunas Str. 30, Babtai 54333, Kaunas reg., Lithuania; inga.tamosiune@lammc.lt; fahad.hakim@lammc.lt; vidmantas.stanys@lammc.lt; jurgita.vinskiene@lammc.lt; elena.andriunaite@lammc.lt

[2] Department of Biochemistry, Vytautas Magnus University, Universiteto Str. 10, Akademija 53361, Kaunas reg., Lithuania; odeta.buzaite@vdu.lt

* Correspondence: danas.baniulis@lammc.lt

Tables

Table S1. Summary statistics of *16S rRNA* gene metataxonomic analysis of the *R. rosea* rhizome samples.

Exp. group	1-year			>4-year		
**Sample**	**A1**	**A2**	**A3**	**B1**	**B2**	**B3**
**Domain**				**V4**		
Number of mapped reads	328,371	137,574	314,767	386,012	369,601	589,600
Number of bacterial reads	24,601	32,587	57,608	4694	4148	6904
**Domain**				**V5-7**		
Number of mapped reads	120,202	108,891	112,163	343,705	241,393	262,233
Number of bacterial reads	7360	45,629	67,948	33,573	218,571	207,900

Table S2. Relative abundance of family level taxa in *Rhodiola rosea* plant rhizome samples. For each experimental group of the 1-year and >4-year-old plants, three plant replicates (indicated as samples A1-3 and B1-3, respectively) were used for DNA extraction. One to four DNA samples isolated from each plant were analysed using *16S rRNA* gene V4 and V5-7 variable region amplicon high-throughput sequencing. The combined data for each plant were mapped using SILVA SSU database. Abundance values higher than 5% and 20% are shown in blue and red font, respectively. (Provided in a separate MS Excel file).

Table S3. Results of bacterial isolate identification using *16S rRNA* gene sequence data.

Age	Name	Bacterial strain	Accession	Seq identity, %	Tax ID
1-year	S1-1	*Pantoea agglomerans*	CP077366.1	98.73%	549
	S1-2	*Peribacillus frigoritolerans*	KU955645.1	99.51%	450367
	S1-3	*Pseudomonas sp.*	OQ221836.1	99.82%	306
	S1-4	*Bacillus toyonensis*	ON933832.1	98.91%	155322
	S1-5	*Lysinibacillus fusiformis*	PQ093568.1	99.45%	28031
	S1-6	*Peribacillus simplex*	MN704413.1	99.09%	1478
	S2-1	*Peribacillus muralis*	MG011552.1	99.54%	264697
	S2-2	*Pantoea agglomerans*	CP077366.1	99.36%	549
	S2-3	*Bacillus* sp.	KF648906.1	98.73%	1399374
	S2-4	*Rahnella aquatilis*	CP036490.1	99.36%	34038
	S3-1	*Bacillus* sp.	JX266343.1	99.45%	1224996
	S4-1	*Bacillus* sp.	KF863884.1	99.54%	1458823
	S4-2	*Serratia* sp.	JN106429.1	99.82%	1054547
	S4-3	*Bacillus cereus*	EU661712.1	99.63%	1396
	S5-1	*Lysinibacillus fusiformis*	PQ093568.1	99.64%	28031
	S5-2	*Bacillus zanthoxyli*	PQ093517.1	99.09%	2663026
	S5-3	*Pseudomonas* sp.	KY907020.1	99.45%	306
	S6-1	*Pseudomonas putida*	PP188569.1	99.27%	303
	S7-1	*Brevundimonas intermedia*	KR085793.1	99.18%	74315
>4-year	L1-1	*Lelliottia amnigena*	MN209789.1	99.26%	61646
	L1-2	*Pseudomonas* sp.	OR363700.1	99.73%	306
	L1-3	*Lelliottia amnigena*	KT767779.1	99.36%	1439331
	L2-1	*Enterobacter* sp.	MK459528.1	99.82%	42895
	L2-3	*Xanthomonas* sp.	FJ405365.1	99.54%	573583
	L3-1	*Klebsiella aerogenes*	EF210101.1	98.66%	548
	L3-2	*Citrobacter* sp.	FN562933.1	80.29%	688073
	L3-3	*Bacillus* sp.	KF831378.1	99.82%	1441669
	L3-4	*Bacillus* sp.	HM566790.1	99.46%	865604
	L3-5	*Enterobacter ludwigii*	OR752044.1	99.82%	299767
	L4-1	*Pseudomonas* sp.	MT165531.1	99.73%	306
	L4-2	*Enterobacter ludwigii*	CP144278.1	98.56%	299767
	L5-1	*Pantoea rodasii*	MN036531.1	95.89%	1076549
	L5-2	*Enterobacter ludwigii*	KR054972.1	99.64%	299767
	L5-3	*Lelliottia nimipressuralis*	ON479624.1	99.09%	69220
	L6-1	*Pseudomonas* sp.	CP054880.1	99.82%	2743971
	L6-2	*Pantoea agglomerans*	CP077366.1	99.54%	1400052
	L7-1	*Bacillus zanthoxyli*	OQ786977.1	99.46%	2663026
	L7-2	*Pantoea* sp.	MW827727.1	98.25%	69393
	L7-3	*Pseudomonas* sp.	CP054880.1	99.91%	2743971
	L7-4	*Pseudomonas* sp.	CP087198.1	100.00%	2895471

Table S3. Results of bacterial isolate identification using *16S rRNA* gene sequence data (continued).

Age	Name	Bacterial strain	Accession	Seq identity, %	Tax ID
>4-years	V1_1	*Rahnella* sp.	NR_181934.1	98.20%	2703885
	V2_1	*Rahnella* sp.	NR_181934.1	97.09%	2703885
	V3_1	*Rahnella* sp.	NR_181934.1	97.78%	2703885
	V4_1	*Rahnella* sp.	NR_180981.1	98.11%	2787622
	V5_1	*Rahnella* sp.	NR_181934.1	98.18%	2703885
	V7_1	*Rahnella* sp.	NR_181934.1	98.35%	2703885
	K1_1	*Bacillus* sp.	NR_164882.1	97.29%	2663026
	D1_1	*Rahnella* sp.	NR_146849.1	98.78%	574964
	D4_1	*Serratia* sp.	NR_157762.1	94.26%	2034155
	D5_1	*Rahnella* sp.	NR_146849.1	98.60%	574964
	D7_1	*Rahnella* sp.	NR_146849.1	98.28%	574964
	D8_1	*Rahnella* sp.	NR_181934.1	96.82%	2703885
	D9_1	*Rahnella* sp.	NR_181934.1	94.20%	2703885
	D9_2	*Pseudomonas* sp.	NR_157778.1	96.25%	1785161
	8-2	*Bacillus* sp.	NR_157731.1	96.36%	2026190
	A5_1	*Bacillus* sp.	NR_102783.2	97.62%	135461
	A9_1	*Erwinia* sp.	NR_119363.1	75.69%	69222
	A1_1	*Bacillus* sp.	NR_104919.1	98.46%	227866
	A3_1	*Bacillus* sp.	NR_181952.1	96.91%	2054641
	A1_B	*Rahnella* sp.	NR_180980.1	97.57%	2703885
	A1_P1	*Pseudomonas* sp.	NR_113600.1	94.39%	47878
	V6_1	*Pseudomonas* sp.	NR_181728.1	97.86%	2833594
	V6_2	*Rahnella* sp.	NR_180981.1	97.97%	2787622
	K2_1	*Erwinia* sp.	NR_104932.1	94.14%	182337
	K2_2	*Rahnella* sp.	NR_180981.1	92.23%	2787622
	K3_1	*Rahnella* sp.	NR_181934.1	87.16%	2703885
	K3_2	*Erwinia* sp.	NR_104932.1	97.08%	634500
	D3_1	*Pseudomonas* sp.	NR_181728.1	98.12%	2833594
	D3_2	*Rahnella* sp.	NR_025337.1	81.48%	745277
	D3_3	*Rahnella* sp.	NR_146849.1	97.75%	574964
	D6_1	*Rahnella* sp.	NR_181934.1	96.85%	2703885
	D6_3	*Pseudomonas* sp.	NR_113651.1	95.47%	1395569
	8-1	*Pseudomonas* sp.	NR_179985.1	96.97%	2697028

Figures

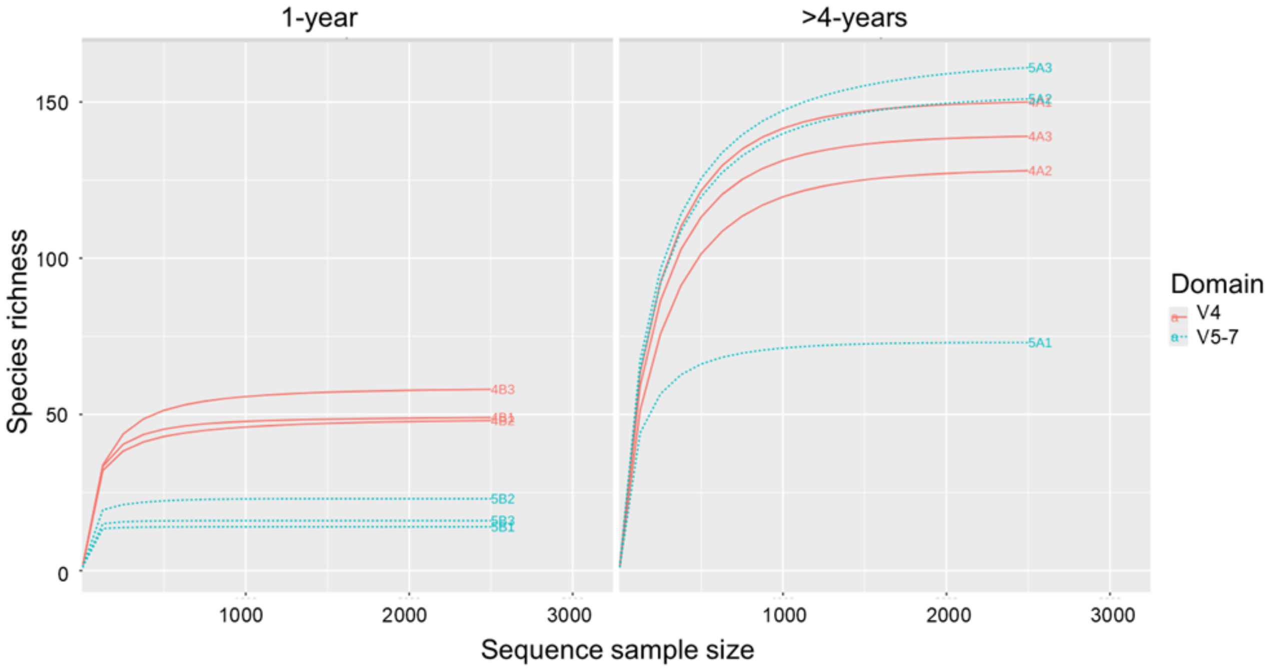

Figure S1. Rarefaction curves for *R. rosea* rhizome samples estimated from *16S rRNA* gene high-throughput sequencing analysis data using the Microbiome Analyst server (Chong et al., 2020 [57]).

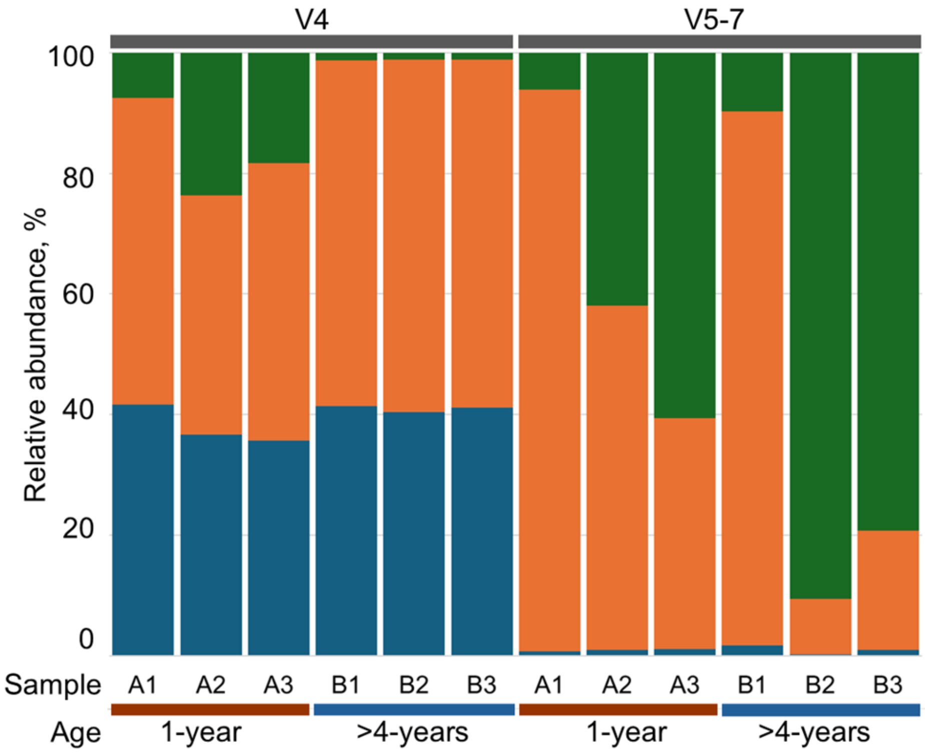

Figure S2. Distribution of the bacterial (red) to plastidial (green) and mitochondrial (blue) reads obtained from *16S rRNA* gene high-throughput sequencing analysis datasets of 1-year and >4-year-old *R. rosea* rhizome samples.

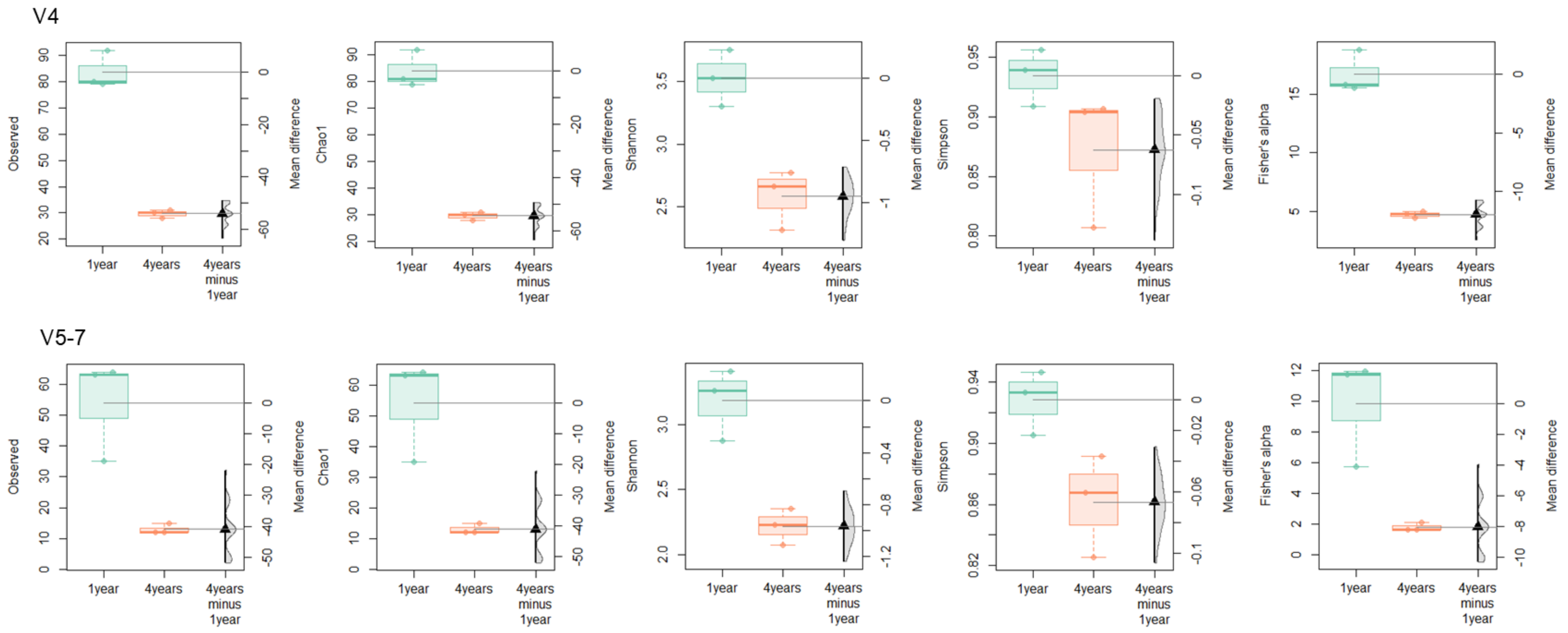

Figure S3. Comparison of alpha diversity indices of *R. rosea* rhizome samples estimated using *16S rRNA* domain V4 (upper panels) and V5-7 (lower panels) amplicon sequencing analysis. Indexes were estimated using the Microbiome Analyst server [57]. Data statistical analysis and visualization was performed using Durga package [58].

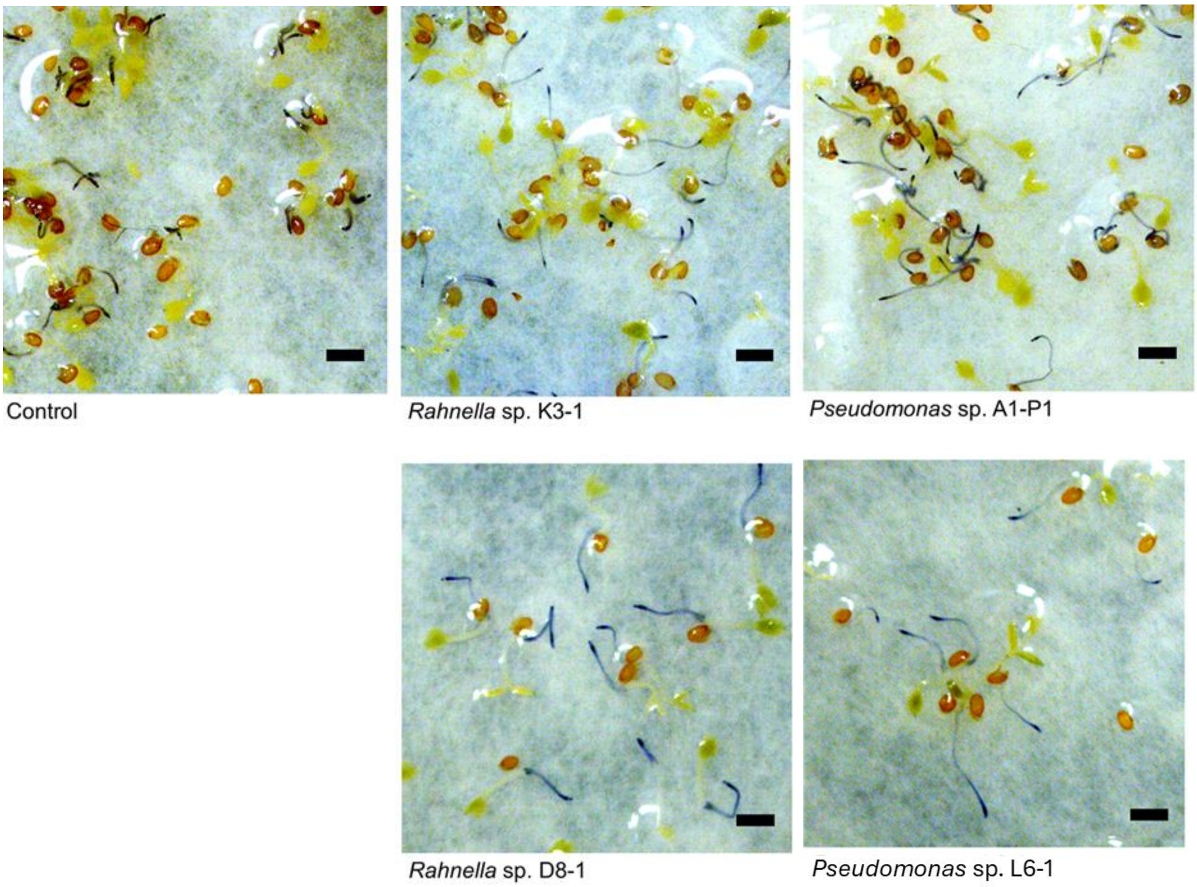

Figure S4. Representative images of Arabidopsis seedling shoot growth-modulating effect of endophytic bacterial isolates obtained from *R. rosea* rhizome. The scale bar represents 1 mm.

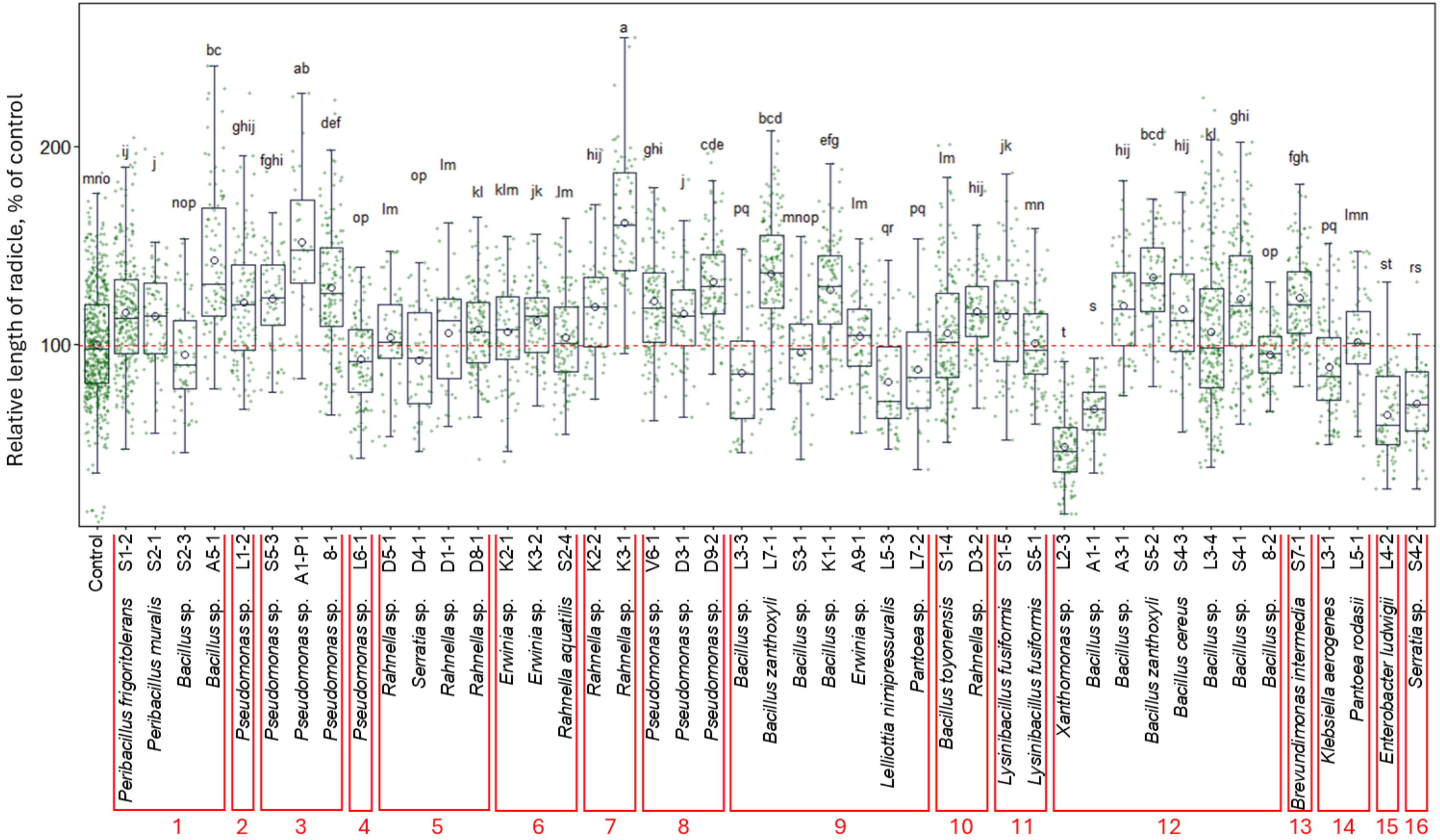

Figure S5. Arabidopsis seedlings root growth-modulating effect of endophytic bacterial isolates obtained from *R. rosea* rhizome. The data are shown as boxplots representing the means, medians, minimum and maximum scores, and lower and upper quartiles; data points are plotted as green dots; the red line indicates the mean value of control; red lines and numbers indicate clusters as shown in Figure 5; different letters denote significant differences between the analysed groups ($p \leq 0.05$).

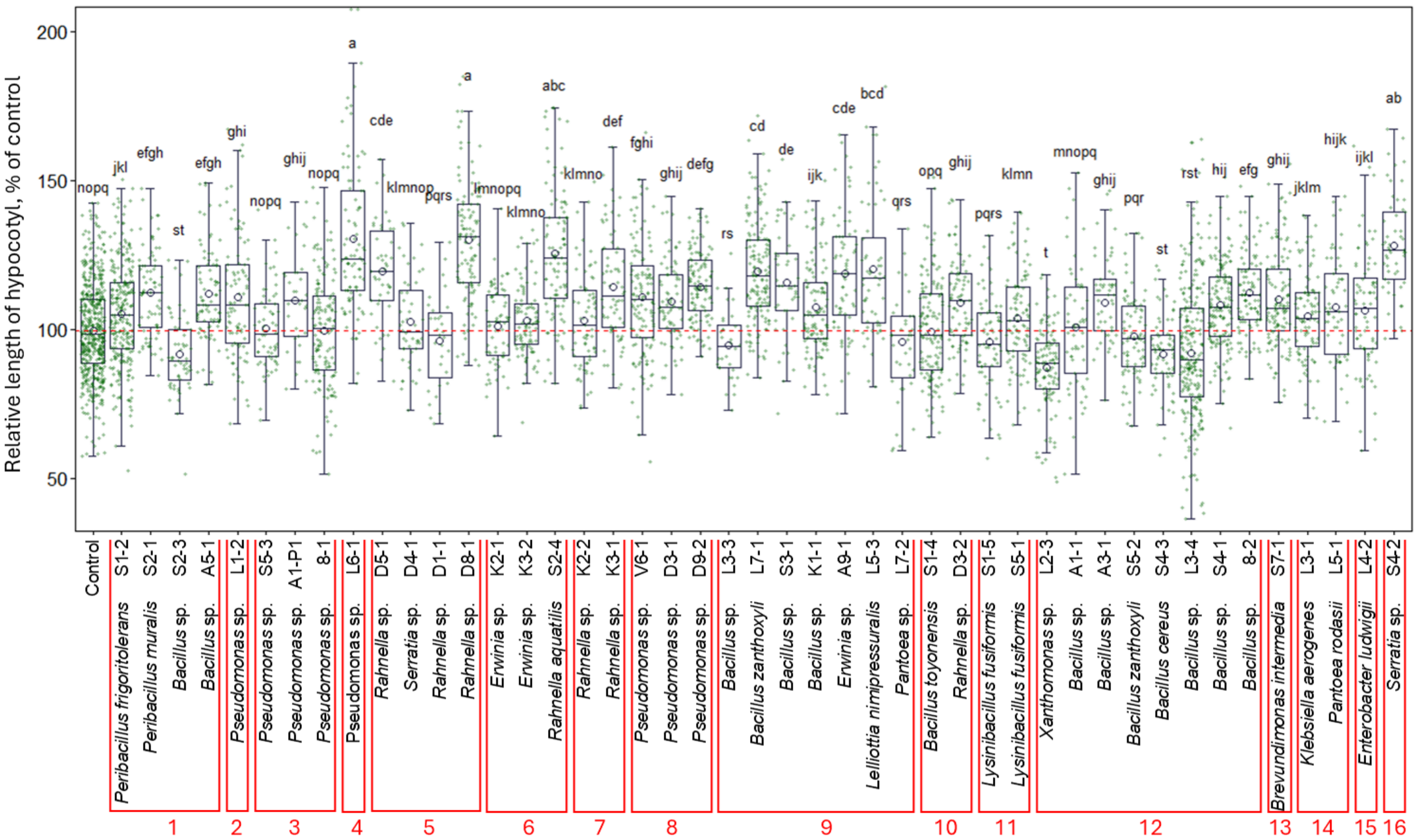

Figure S6. Arabidopsis seedlings shoot growth-modulating effect of endophytic bacterial isolates obtained from *R. rosea* rhizome. The data are shown as boxplots representing the means, medians, minimum and maximum scores, and lower and upper quartiles; data points are plotted as green dots; the red line indicates the mean value of control; red lines and numbers indicate clusters as shown in Figure 5; different letters denote significant differences between the analysed groups ($p \leq 0.05$).
